# Crisis-focused Cognitive Behavioural Therapy for psychosis (CBTp) in acute mental health inpatient settings (the CRISIS study): protocol for a pilot randomised controlled trial

**DOI:** 10.1186/s40814-022-01160-7

**Published:** 2022-09-10

**Authors:** Lisa Wood, Claire Williams, Vanessa Pinfold, Fiona Nolan, Anthony P. Morrison, Nicola Morant, Brynmor Lloyd-Evans, Glyn Lewis, Barbara Lay, Rebecca Jones, Kathryn Greenwood, Sonia Johnson

**Affiliations:** 1 Division of Psychiatry, University College London, 149 Tottenham Court Road, London, W1T 7NF UK; 2 Acute and Rehabilitation Directorate, North East London NHS Foundation Trust, Goodmayes Hospital, Barley Lane, Ilford, IG3 8XJ UK; 3 McPin Foundation, 7-14 Great Dover St, London, SE1 4YR UK; 4 Health Education England, 32 Russell Square, London, WC1B 5DN UK; 5 Psychosis Research Unit, Greater Manchester Mental Health NHS Trust, Harrop House, Prestwich Hospital, Bury New Road, Manchester, M25 3BL UK; 6 University of Manchester, Zochonis Building, Manchester, M13 9GB UK; 7 Psychiatrische Dienste Aargau AG, Klinik für Psychiatrie und Psychotherapie, Evaluation Lehre und Forschung, Königsfelderstrasse 1, 5210 Windisch, Switzerland; 8 School of Psychology, University of Sussex, Pevensey Building, Falmer, BN1 9QH UK; 9 Sussex Partnership NHS Foundation Trust, Worthing, Sussex BN13 3EP UK

**Keywords:** Randomised controlled trial, Crisis intervention, Inpatients, Psychosis, Cognitive behavioural therapy, CBTp

## Abstract

**Background:**

Cognitive Behavioural Therapy for psychosis (CBTp) has an established evidence base and is recommended by clinical guidelines to be offered during the acute phases of psychosis. However, few research studies have examined the efficacy of CBTp interventions specifically adapted for the acute mental health inpatient context with most research trials being conducted with white European community populations.

**Aims:**

The aim of this study is to conduct a pilot randomised controlled trial (RCT), which incorporates the examination of feasibility markers, of a crisis-focused CBTp intervention adapted for an ethnically diverse acute mental health inpatient population, in preparation for a large-scale randomised controlled trial. The study will examine the feasibility of undertaking the trial, the acceptability and safety of the intervention and the suitability of chosen outcome measures. This will inform the planning of a future, fully powered RCT.

**Methods:**

A single-site, parallel-group, pilot RCT will be conducted examining the intervention. Drawing on principles of coproduction, the intervention has been adapted in partnership with key stakeholders: service users with lived experience of psychosis and of inpatient care (including those from ethnic minority backgrounds), carers, multi-disciplinary inpatient clinicians and researchers. Sixty participants with experience of psychosis and in current receipt of acute mental health inpatient care will be recruited. Participants will be randomly allocated to either the crisis-focused CBTp intervention or treatment as usual (TAU).

**Discussion:**

Findings of this pilot RCT will indicate whether a larger multi-site RCT is needed to investigate the efficacy of the intervention. If the initial results demonstrate that this trial is feasible and the intervention is acceptable, it will provide evidence that a full-scale effectiveness trial may be warranted.

**Trial registration:**

This trial has been prospectively registered on the ISRCTN registry (ISRCTN59055607) on the 18th of February 2021.

## Introduction

Cognitive Behavioural Therapy for psychosis (CBTp) is a psychological therapy which targets the distress relating to experiences of psychosis by adapting the way an individual thinks and behaves. Although there have been recent reviews challenging the effectiveness of CBTp [21, 22], the evidence base generally demonstrates that CBTp is an effective intervention, with a small to medium effect size, in improving outcomes for people experiencing psychosis [46, 52]. CBTp is recommended by clinical guidelines, such as the National Institute of Health and Clinical Excellence [30], to be offered in the acute phase of psychosis, i.e. in inpatient settings. However, despite this recommendation, the majority of the research examining the effectiveness of CBTp has been done with outpatient community samples or in outdated inpatient settings. These settings are not reflective of the current inpatient context, which have a higher threshold for admission and care for more ethnically diverse service users [19, 49].

It is well demonstrated that the acute stage is an important time to intervene psychologically as this is when people are in most need of psychological support; are often at risk to themselves (e.g. self-harm or suicide), to (e.g. violence and aggression) and/or from (e.g. exploitation) others; and are experiencing distressing symptoms of psychosis [45]. The average stay on an acute mental health inpatient ward in England is 31 days [28]; therefore, there is ample time to offer a psychological intervention which is brief, targeted to the crisis and adapted to meet the needs of this population [7]. There is evidence that cognitive behavioural interventions can be adapted to be delivered as a brief intervention, i.e. within 6–8 sessions [14, 41], and are suitable for inpatients [41]. Therefore, an inpatient admission may be a time where people could engage in and derive benefit from a psychological intervention [11].

There is an overrepresentation of inpatient service users from ethnic minority backgrounds, with those from black African and black Caribbean backgrounds being four times more likely to be compulsorily detained compared to their white counterparts [29]. There is evidence that service users from ethnic minority backgrounds are less likely to be offered psychological therapies, have more negative experiences of mental health services [17] and have poorer outcomes following a mental health crisis [11]. Therefore, it is essential that acute inpatient psychological interventions are also developed to meet the needs of ethnic minority groups to be useful and acceptable in this setting. There is some existing evidence that CBTp can be culturally adapted to be appropriate for ethnic minority populations; however, this research has not been undertaken with inpatient populations [15, 37].

Three recent systematic reviews have been conducted to examine the feasibility, acceptability and effectiveness of cognitive behavioural psychological interventions for people in acute mental health inpatient settings [19, 34, 49]. They have demonstrated some initial promise with small improvements in psychotic symptoms at the end of therapy, but not at longer-term follow-up [34, 49]. There is also initial evidence that psychological therapy may have a small effect on readmission, depression and anxiety [19]. However, all reviews agreed that the quality of research was poor to moderate and that the majority of psychological therapies had not been specifically adapted for delivery in the acute mental health inpatient setting, or focused on the current mental health crisis [49]. This is a strong indication of a need for a robust evaluation of a psychological intervention, such as CBTp, adapted for the current acute mental health inpatient setting with a focus on crisis reduction.

An adapted crisis-focused CBTp intervention for inpatient settings, which is culturally competent, has been developed following best practice guidelines from the Medical Research Council (MRC) on complex intervention development [42]. The CBTp intervention was developed from several sources of information including a systematic review [49], relevant psychological theory [8, 26], core competency frameworks for working with people experiencing psychosis [38], and acute mental health inpatient care respectively [51], three qualitative interview studies [47, 48, 50], a Delphi study [27] and additional stakeholder consultation as outlined in the methodology.

The aim of this study is to conduct a pilot randomised controlled trial, which includes examination of feasibility markers, of the outlined adapted crisis-focused CBTp intervention for acute inpatients experiencing psychosis. More specifically, this study will:

1. Assess the feasibility of conducting the RCT (examined by the number of service users who give informed consent as a proportion of the number identified as eligible, recruitment timeframe, the number of service users randomised, number of participants who drop out from the trial, number of post-therapy and follow-up assessments completed and participant qualitative feedback regarding the research process).
2. Examine the acceptability of the intervention:
  - To service user participants (examined by the number of therapy sessions attended, number of sessions declined or not attended, number of people who declined therapy once randomised, therapy modules/resources used, mode of therapy, location of therapy sessions, number of sessions conducted prior to discharge, time and duration of sessions, qualitative feedback on the intervention)
  - To therapists (examination by therapists’ fidelity, therapist experience of the training intervention and delivery of therapy)
3. Examine the safety of the intervention through the monitoring of adverse events
4. Examine the suitability of the outcome measures to examine the efficacy of the interventions, in preparation for a fully powered effectiveness RCT

## Methodology

### Design

**Fig. 1:**
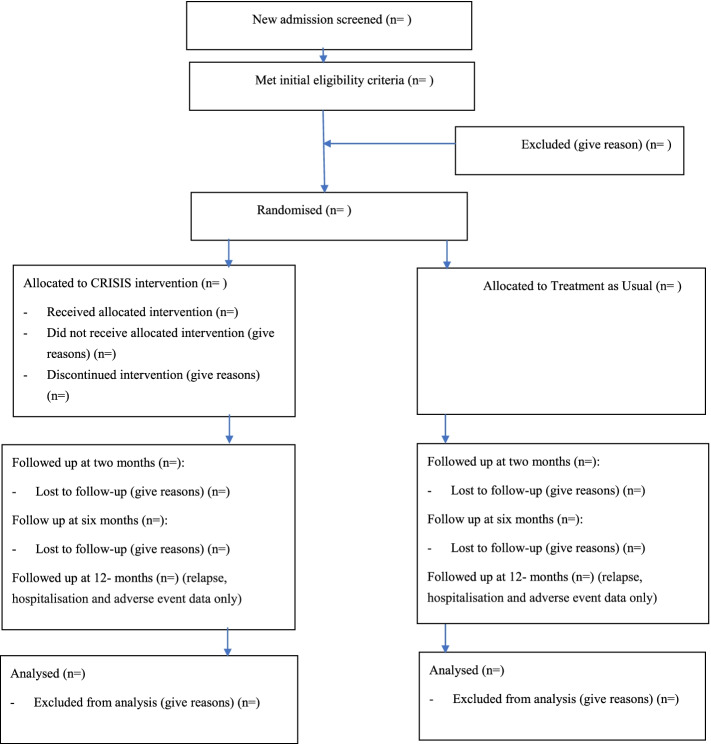
Diagram of the study flow

This study will adopt a single-centre, individually randomised, parallel-group, RCT design with an embedded qualitative component. Participants will be randomly allocated to either treatment as usual (TAU) or CBTp plus TAU in a 1:1 ratio, as undertaken in other psychological therapy trials conducted in inpatient settings [20, 36]. Outcome measure assessments will be carried out at baseline, 2 months post-randomisation and 6 months post-randomisation either face to face or remotely. Relapse, rehospitalisation and adverse event data will be again examined 12 months post-randomisation. This protocol is reported following guidance from the SPIRIT tool (Standard Protocol Items: Recommendations for Intervention Trials, [10]). The trial has been registered on the ISRCTN trial registry (ISRCTN59055607). The study plan is outlined in Fig. 1 and the schedule of participant activities is in Table 1. Full Health Research Authority (HRA) and NHS Research Ethics Committee (REC) approval has been granted (IRAS ID: 272043; 20/LO/0137/AM01) and the study is sponsored by the University College London.

**Table 1:** Schedule of events

	Enrolment	Baseline	Allocation	2-month FU	6-month FU	12-month FU
**Eligibility screen**	**X**					
**Informed consent**	**X**					
**Intervention allocation**			**X**			
**Outcomes**						
PANSS-P		**X**		**X**	**X**	
BDI-7		**X**		**X**	**X**	
BHS-9		**X**		**X**	**X**	
GAD-7		**X**		**X**	**X**	
QPR		**X**		**X**	**X**	
CRISP		**X**		**X**	**X**	
GAF		**X**		**X**	**X**	
TAG		**X**		**X**	**X**	
Relapse				**X**	**X**	**X**
Rehospitalisation				**X**	**X**	**X**
Adverse events^a^				**X**	**X**	**X**
**Qualitative interviews**^b^				**X**		

*BDI-7* Beck Depression Inventory, *BHS-9* Beck Hopelessness Scale, *CRISP* Crisis Scale in Psychosis, *GAD-7* Generalised Anxiety Disorder Measure, *GAF* Global Assessment of Functioning, *PANSS-P* Positive and Negative Syndrome Scale—Positive Subscale, *QPR* Process of Recovery Questionnaire, *TAG* Threshold Assessment Grid, *FU* follow-up

^a^Adverse events will be monitored at each time point and at every therapy session

^b^Will occur 4–8 weeks post-intervention completion

### Setting

This trial will be undertaken at a single NHS site, North East London NHS Foundation Trust in the acute mental health inpatient services.

### Sample size

We will aim to recruit sixty participants in line with recommendations by Consolidated Standards of Reporting Trials (CONSORT) for feasibility and pilot RCTs [13], thirty of which will be randomly allocated to the therapy arm and thirty to the TAU arm. A sample of sixty is deemed of sufficient size to examine the aims of the study. Data (relevant means and standard deviations) from this study will be used to inform a power calculation for a future larger multi-site trial. The sample size will also allow for dropouts, which are common in clinical trials in acute mental health inpatient settings. Some studies report attrition rates of over 50% [36], and recruiting a larger sample will still allow us to collect adequate data to inform a larger multi-site trial.

### Participants

Purposive sampling will be conducted to ensure that the sample is representative of the service user population, with at least 50% from ethnic minority backgrounds and at least 30% of the total sample from Black ethnic backgrounds.

Inclusion criteria for participants will be (i) aged 18 and above; (ii) who meet criteria for a schizophrenia-spectrum diagnoses (schizophrenia, schizophreniform disorder, schizoaffective disorder, delusional disorder or psychotic disorder not otherwise specified; ICD-10), or meet criteria for an early intervention service (EIS) for treatment of psychosis to allow for diagnostic uncertainty; (iii) able to give informed consent and have the capacity to consent; (iv) currently receiving care from an acute psychiatric inpatient team; and (v) able to complete the research in English. Exclusion criteria will be (i) non-English speakers (due to translation costs and difficulty of producing valid translations of the research instruments and intervention), (ii) an acquired brain injury or substance misuse judged to be the acute cause of the psychotic experiences and (iii) those already undertaking a structured psychological intervention delivered by a psychologist or trained therapist at the time of the study.

### Randomisation and blinding

The randomisation will be undertaken using www.sealedenvelope.com. Participant numbers will be entered into the sealed-envelope website by an individual independent from the trial after the research assistant has completed the baseline assessment. Allocations will be emailed to the PI who will then contact the therapists to commence therapy with the participants assigned to the intervention arm and inform participants of their allocation.

All assessments will be completed by research assistants who will be blind to treatment allocation. The research assistants will work in a separate team and building with the therapists to minimise blind breaks. The research assistants will be appropriately trained to administer all outcome measures. Blinding will be monitored, and if any blind breaks (i.e. the research assistant being exposed to the participant’s treatment condition) occur, they will be systematically recorded.

### Stakeholder involvement for the development of this protocol

This research protocol was presented at the North Thames Collaborations for Leadership in Applied Health Research and Care (CLAHRC) Patient and Public Involvement (PPI) panel to shape study conception and research design (twice in 2018). The panel supported the aims of this project and thought the outlined methodological design was appropriate and recommended the development of a stakeholder consultation group who are consulted throughout the duration of the research study (particularly during the intervention development). The panel also gave some initial feedback on the core components of the intervention which were all integrated into the therapy protocol.

### Stakeholder involvement for this research study

A stakeholder group of eleven experts (service users, family/carers, inpatient clinicians and researchers) was convened in August 2020 to contribute to key decisions about the design of the intervention and research trial and was informed of coproduction principles outlined by INVOLVE [18] and the National Survivor User Network [32] 4pi framework. Recruitment for the panel was conducted through the McPin Foundation and the Mental Health Policy Research Unit (MHPRU) respectively for service user and carer stakeholders, and through social media and the professional network of the lead researcher respectively for mental health inpatient staff and researchers. Purposive recruitment of stakeholders was undertaken to ensure there was diversity within the group. Meetings were conducted every month whilst the intervention was being developed and will continue at quarterly intervals for the duration of the trial. Six stakeholder meetings have been undertaken to date (August to January 2020) and have focused on the adaptions to the CBTp intervention including identifying the key underlying values and principles, delivery format, crisis priorities for service users and carers, how to include family/social networks, culturally appropriate adaptations (e.g. including religious spiritual and community understanding and support into the intervention) and ensuring cultural sensitivity. To date, all meetings have been conducted remotely due to COVID-19.

### Study arms

Participants will be randomised to one of two conditions: the crisis-focused CBTp intervention arm or the treatment as usual arm.

#### Crisis-focused CBTp intervention

The trial intervention will be delivered by psychological therapists working in the acute inpatient services. All therapists are clinical psychologists or counselling psychologists registered with the UK Health and Care Professions Council [HCPC], with experience of delivering CBTp in inpatient settings.

The crisis-focused CBTp intervention is underpinned by a modularised CBTp protocol that aims to deliver approximately six to eight sessions of therapy to participants. The protocol includes seven modules; engagement, assessment and identifying priorities; formulation of the crisis; stabilisation and safety; coping, self-management and problem solving; crisis plans and crisis cards; change strategy work focusing on crisis appraisals and safety behaviours; and discharge, relapse planning and recovery toolkits. The first two modules (engagement, assessment and identifying priorities, and formulating the crisis) are essential components of the intervention and the remaining ones are collaboratively chosen based on the participant’s priorities. The number of sessions will be determined by the collaborative priorities set by the service user and therapist and the length of admission, and therefore, more or less sessions can be offered. The therapy will also include at least one follow-up session post-discharge to ensure support through the discharge process. The intervention will also include strategies to involve the individual’s network, e.g. family sessions, and formulation sharing with the multi-disciplinary team.

The sessions will be delivered in a private and quiet room on the inpatient ward or outside/off the ward if the participant has the appropriate leave, e.g. in a room off the ward but on the grounds of the hospital. If the participant is discharged before therapy is complete, the sessions will continue and be delivered in community settings or remotely (e.g. via phone or video conferencing technology). This is to ensure that there is continuity in sessions post-discharge.

##### Training and supervision of the therapists

The therapists received a training package which involved watching pre-recorded therapy videos (https://www.psychosisresearch.com/cbt-phase-1/), and 2 days of training specifically focusing on crisis-focused CBTp. The training, delivered by LW, CW and a service user with lived experience of therapy and inpatient care, comprised an introduction to the crisis-focused CBTp model, making culturally appropriate adaptations, undertaking a crisis-focused assessment, developing a crisis-focused formulation and utilising brief crisis-focused intervention strategies. It included a combination of didactic teaching, role plays, reflective exercises and group discussion. They will also have access to weekly 90-min group supervision whilst delivering the therapeutic intervention.

##### Fidelity to therapy

All therapy sessions will be audio recorded, if the participants consent. Ten percent will be rated on the Cognitive Therapy Rating Scale [5] to ensure adherence to the CBT model. Some adaptations to the application of the fidelity scale will be made, taking into account that the therapy is being delivered as a brief intervention and with people in an acute mental health inpatient population, which may make some of the fidelity items harder to achieve. For example, the agenda may only be brief and only include a single agenda item, specific thoughts/cognitions may not always be identifiable, and only a simple formulation may be possible (making basic links between thoughts, feelings and behaviours).

##### Monitoring what is delivered

The therapists will record the content and delivery of the sessions, including information on the session length, agenda and types of CBTp strategies utilised (e.g. assessment, formulation, relapse prevention) on a pre-defined database.

#### Treatment as usual (TAU)

This will be the routine care that participants receive within the acute inpatient setting. This includes multi-disciplinary care from mental health nurses, nursing assistants, psychiatrists, pharmacists, occupational therapists and psychologists. TAU will include access to routinely delivered psychosocial intervention; this can include structured psychological therapies delivered by clinical or counselling psychologists and brief interventions delivered by appropriately trained nurses or occupational therapists. The NHS study site employs a maximum of 0.4 whole time equivalent (WTE) of a qualified psychologist (clinical or counselling) and 0.8 WTE of an assistant psychologist input per 20 acute care beds. The trial therapists also work in treatment as usual; however, recruitment will not be conducted on the ward in which they are offering treatment as usual so it is extremely unlikely study participants will be offered therapy from the trial therapists. They will also not use any of the therapy resources in treatment as usual.

### Procedure

Participants will be recruited from three male acute wards, three female acute wards, three acute wards for older adults and one male psychiatric intensive care unit (PICU) in North East London NHS Foundation Trust (NELFT). The study will be advertised to the inpatient staff teams who will in turn provide information about the study to eligible participants. Due to the high turnover in acute mental health inpatient wards, inpatient staff will advertise the research to service users as early as the first day on the ward, if appropriate to do so. If participants consent, they will be contacted by a research assistant within 72 h who will share more detailed information and gain informed consent if the service user wants to participate. The research assistant will undertake all clinician-administered and self-report measures with the participant (see Table 1). Once complete, the research assistant will inform the PI of the participant number who will randomise the participant to either the CBTp intervention or TAU. The PI will inform the participant of their allocation. The research assistant will then complete further assessment sessions with all participants at 2 months post-randomisation and 6 months post-randomisation. Relapse, rehospitalisation, and adverse event data will be collected at 12 months, which does not involve contact with the participant.

### Outcomes

#### Feasibility outcomes

Individual patient data will be collected on a pre-developed sheet adhering to CONSORT guidelines [6, 39]. This will include the patient eligibility status, number of referrals received by ward staff, willingness of participants to consent, willingness of participants to be randomised and attrition. Therapy-specific data will include the number of sessions attended, number of sessions declined or not attended, number of people who declined therapy once randomised, therapy resources used, mode of therapy, location of therapy sessions, number of sessions conducted prior to and post-discharge, time and duration of sessions, quantity of homework completed and any serious adverse events. Patient outcome measure completion will also be examined.

An individual patient demographics sheet will be used to collect sociodemographic and service user data, in order to identify the types of people willing to take part in such a trial.

#### Clinical outcomes

Clinical outcome measures will be completed by a research assistant blind to treatment allocation. In order to reduce participant burden, a combination of self-report outcomes, assessor-rated outcomes and a structured clinical interview has been chosen. The measures will be administered during a face-to-face, or if not possible remote, interview between the participant and the research assistant. Where possible, short versions or sub-components of the measures have been chosen for the same reason.

##### Semi-structured clinical interview

- Experiences of psychosis will be measured by the Positive and Negative Syndrome Scale (PANSS) [23]. The PANSS is a frequently used measure of psychosis and has three subscales of positive symptoms, negative symptoms and general pathology. Only the positive subscale will be used to measure positive symptoms. It is a 7-item subscale which will be completed by trained research assistants. Participants can score from 1 (absent) to 7 (extreme) on each item. It has good internal consistency (Cronbach’s *α* = .73) and inter-rater reliability (between 0.83 and 0.87).

##### Self-report outcome measures (to be completed with assistance from the researcher)

- Depression will be measured by the Beck Depression Inventory brief 7-item measure (BDI-7 [3]). This is a self-report measure which has good internal consistency. Participants can score from 0 (not present) to 3 (indicating severe) and have a total score of 21. Items examine symptoms of sadness, pessimism, past failure, loss of pleasure, self-dislike, self-criticism and suicidal thoughts. It has good internal consistency (Cronbach’s *α* = .86).
- Hopelessness will be measured by the short-form of the Beck Hopelessness Scale (BHS; [2]), which is a recently developed 9-item version of the scale validated for psychiatric inpatients [1]. The 9-item measure includes items 2, 6, 11, 12, 14, 16, 17, 18 and 20 from the original scale. Participants can score 0 (not present) or 1 (present) on each item. Items include “My future seems dark to me” and “I have great faith in the future”. The 9-item version has good internal consistency (Cronbach’s *α* = .86).
- Personal recovery will be measured by the 15-item Process of Recovery Questionnaire (QPR; [25]). Participants can score from 0 (disagree strongly) to 4 (agree strongly) on each item and score a total maximum of 40 on the scale. Items include “I feel better about myself” and “I feel that my life has a purpose”. It has good internal consistency (Cronbach’s *α* = .93).
- Anxiety will be measured on the Generalised Anxiety Disorder-7 item (GAD-7) [44] measure. Participants can score from 0 (not at all) to 3 (nearly every day) on each item. A potential total score can range from 0 to 21. It has good internal consistency (Cronbach’s *α* = .92).
- Subjective experiences of crisis in psychosis will be examined using a newly developed measure (Wood et al, unpublished). This is a 23-item measure which includes statements such as “I have been feeling very strong distressing emotions” and “I feel able to live my life in line with my morals and values” with scores of between 1 (not at all) and 5 (all of the time) on each item. Participants can score a total of 115. This is not yet validated but its psychometric properties will be examined using study data.
- Quality of life will be measured by the Recovering Quality of Life (REQOL-10; [23]). Participants rate their quality of life on 10 items from 0 to 4. A participant can score a total of 40. Example items include “I felt lonely” and “I felt happy”. Internal consistency is high (Cronbach *α* = .89).
- Service use will also be assessed using an adapted version of the “generic UK mental health” version of the Client Service Receipt Inventory (CSRI; [4]). This has been adapted to reflect the local care pathways in the recruitment site. This will be completed through participant self-report and information from clinical notes.

##### Clinician/researcher-rated measures

- Functioning will be assessed on the Global Assessment of Functioning (GAF) measure [16, 35]. GAF scores range from 1 (in some danger of hurting self or others) to 100 (absent or minimal symptoms), examining both symptoms and functioning. It has demonstrated good internal consistency (Cronbach’s *α* = .80).
- The Threshold Assessment Grid (TAG) examines the severity of participants’ mental health difficulties [43]. It has seven domains measuring three areas of safety, risk and needs and disabilities. Participants can score from 0 (none) to 4 (very severe) on each domain, which is analysed individually/as a total score. It has good internal consistency (Cronbach’s *α* = .70).

Participants’ clinical records will be examined to gather information on:

- Hospitalisation: Total number of days in hospital and number of incidents
- Relapse: Total days under the care of acute mental health services (including inpatient wards, crisis home treatment, psychiatric liaison and acute crisis and assessment teams) and number of incidents
- Number of serious incidents and adverse events, including those of harm to self and others

### Qualitative interviews

Qualitative interviews will be conducted to explore service users’ and therapists’ experience of the crisis-focused CBTp intervention and research trial more broadly. This is to evaluate the acceptability of the therapy and therapists’ training.

Participants in the CBTp arm will be invited to complete a qualitative semi-structured interview exploring their experiences of the intervention and its perceived impacts. A purposive sampling approach will be undertaken to ensure a representative sample of participants. We will make efforts to ensure we recruit an ethnically diverse sample and include those who dropped out from therapy. The interview guide will include questions focusing on acceptability following guidance from Sekhon et al. [40] on examining acceptability including how they felt about the intervention, levels of burden, intervention coherence, perceived effectiveness and how it fitted with their value system. The interviews will be undertaken approximately 4 to 8 weeks post-intervention. We aim to interview half of the participants in the CBTp arm; therefore, an estimated total of *n* = 12–15 participants, depending on the quality, depth and diversity of data collected, as this is recommended as an adequate sample size for qualitative interviews analysed by thematic analysis [9]. A purposive sampling approach will be undertaken to ensure a representative sample of participants. 

Semi-structured interviews will also be conducted with the therapists delivering the CBTp intervention and examine perceived acceptability again following guidance from Sekhon et al. [40]. The sample size will be dependent on the number of therapists recruited to deliver therapy but it is estimated that this will be approximately four to six participants. The interview topic guide will include questions on the therapists’ impressions on the usefulness of the training, positive and negative aspects of the training and areas for development. The interview guide includes questions on therapists’ impressions of the usefulness of therapy, expectations and goals, positive and negative aspects of CBTp and relationship with the service user, and suggestions for treatment refinement. This is to evaluate the feasibility and acceptability of the training. The interviews will be undertaken once the therapist has completed all of their allocated therapy cases.

### Analysis plan

The study will follow best practice guidance for the reporting of feasibility RCTs (CONSORT, [13, 39]). The focus of the analysis will be on key indicators of feasibility, including participant recruitment, retention and acceptability of the intervention, which will be summarised descriptively using frequencies and percentages. Continuous clinical outcome measures will be summarised separately by study arm using means and standard deviations or medians and interquartile ranges, as appropriate for the distribution of the data. Binary outcome measures will be summarised using frequencies and percentages. The quantity of missing data for each clinical outcome will be examined and likewise summarised by study arm. This pilot study will not have sufficient power to assess the effectiveness of the intervention. However, to trial the analysis envisaged for a future, fully powered, effectiveness RCT, clinical outcomes at follow-up time points will be compared between study arms, using linear or logistic regression models as appropriate, and adjusting for the baseline measure of the outcome in question.

The qualitative interviews will be audio recorded, transcribed verbatim and analysed using thematic analysis [9] by LW on NViVo [33]. The thematic analysis will be conducted from a critical realist position and will combine inductive and deductive approaches, seeking both to explore participants’ experiences of receiving or delivering CBTp and to answer questions about the acceptability and feasibility of the therapy, its components and its mode of delivery. Initially, each transcript will be read and reread to ensure the researcher is fully immersed in the data, and then initially coded. Codes will be collated together across interviews and grouped together to form analytical themes. Patterns of themes will be explored across the data set focussing on both commonalities and variations and comparing service user and therapist perspectives. The theme structure will be checked with a small number of participants to check it reflects their experiences. The final theme structure will also be discussed with the research team and stakeholder group (including people with lived experience of psychosis and inpatient admission).

#### Feasibility criteria

The trial of the CBTp intervention will be deemed feasible if the following criteria, determined by examining published CBTp trials, are met [19, 49]:

- Retention rate of 75% at 2 months post-randomisation and 60% at 2 months post-randomisation
- Recruitment of ≥80% of the target sample size (*n* = 60) over the 12-month recruitment period
- Qualitative data from service users and therapists support the intervention/indicate that the intervention is acceptable.

### Data management and security

Data will be managed in line with the General Data Protection Regulation 2016/679. All personal identifiable data will be stored separately to study data so data remains anonymous. All participants will be allocated a participant number. All paper documentation will be stored in a locked filing cabinet in a locked office on the NHS site. All electronic data will be stored in a password-protected database and on an encrypted laptop. All audio recordings will be identifiable by participant number and stored on an encrypted laptop.

### Data quality

All data collected through outcome measures and clinical notes will be routinely audited and cross-checked to ensure accurate collection and entry.

### Study governance and management

The CI will manage the study and have responsibility for the trial data. The CI will have monthly meetings with primary collaborators, and research assistants respectively, to ensure the appropriate management of the trial. The sponsor and the REC will be provided with relevant study data for auditing and review processes.

### Trial Steering Committee

A Trial Steering Committee (TSC) has been established which will incorporate the duties of a Data Management Committee (DMC) including monitoring adverse events and monitoring data. The TSC comprises four members with collective expertise in trial statistics, conducting trials, delivering inpatient psychological therapy and lived experience of acute mental health inpatient care. The committee will monitor the progress, conduct and safety of the trial; advise on scientific credibility; and consider if the trial should continue, be modified or be stopped [31]. The group will meet every 6 months whilst the trial is undertaken.

### Dissemination

This trial will be published in a peer-reviewed journal and data will be made available via the Open Science framework. Findings will also be made available to participants and teams where recruitment was conducted and be presented at relevant national and international conferences.

### Trial status

Recruitment commenced on the 18th of February 2021 and will be open until May 2022.

## Discussion

The protocol has outlined a study to assess the feasibility of measures and methods to evaluate an adapted CBTp intervention in acute mental health inpatient settings, the findings of which will inform the feasibility and design of a larger, adequately powered trial. This study will provide useful information on the feasibility of undertaking the trial and the acceptability of the intervention. The intervention has been adapted for use with people in this setting, including those from ethnic minority backgrounds, thus responding to an important priority to improve inpatient care for over-represented inpatient groups [28]. Moreover, there is a need to develop acceptable and feasible interventions for this population and reduce their readmission rates, which is a UK government priority [12]. This trial will contribute to addressing this priority.

## Data Availability

The datasets during and/or analysed during the current study are available from the corresponding author on reasonable request.
